# The *C. elegans* Crumbs family contains a CRB3 homolog and is not essential for viability

**DOI:** 10.1242/bio.201410744

**Published:** 2015-02-06

**Authors:** Selma Waaijers, João Jacob Ramalho, Thijs Koorman, Elisabeth Kruse, Mike Boxem

**Affiliations:** Division of Developmental Biology, Department of Biology, Faculty of Science, Utrecht University, Padualaan 8, 3584 CH, Utrecht, The Netherlands.

**Keywords:** *C. elegans*, cell polarity, Crumbs, CRB

## Abstract

Crumbs proteins are important regulators of epithelial polarity. In *C. elegans*, no essential role for the two described Crumbs homologs has been uncovered. Here, we identify and characterize an additional Crumbs family member in *C. elegans*, which we termed CRB-3 based on its similarity in size and sequence to mammalian CRB3. We visualized CRB-3 subcellular localization by expressing a translational GFP fusion. CRB-3::GFP was expressed in several polarized tissues in the embryo and larval stages, and showed apical localization in the intestine and pharynx. To identify the function of the Crumbs family in *C. elegans* development, we generated a triple Crumbs deletion mutant by sequentially removing the entire coding sequence for each *crumbs* homolog using a CRISPR/Cas9-based approach. Remarkably, animals lacking all three Crumbs homologs are viable and show normal epithelial polarity. Thus, the three *C. elegans* Crumbs family members do not appear to play an essential role in epithelial polarity establishment.

## INTRODUCTION

Cell polarity is of vital importance for the proper development and functioning of epithelial tissues. Epithelial cells are polarized into distinct apical and basolateral plasma membrane domains, separated by the apical junctional complex (AJC). Studies in *Caenorhabditis elegans* and *Drosophila melanogaster* identified three evolutionarily conserved groups of proteins that control the establishment and maintenance of apical and basolateral membrane domains (St Johnston and Ahringer, 2010). Members of the Scribble group (SCRIB/DLG/LGL) localize to the basolateral side and promote basolateral identity, while the apically localized PAR (PAR-3/PAR-6/aPKC) and Crumbs (CRB/PALS1/LIN-7/PATJ) complexes define apical identity.

The Crumbs protein was originally identified in *Drosophila*, where it plays an important role in the establishment of epithelial polarity, specification of apical membrane identity, and the formation of adherens junctions (AJs) (Tepass, 2012). In addition, Crumbs may contribute to the control of tissue growth by regulating the Hippo and Notch signaling pathways (Chen et al., 2010; Grusche et al., 2010; Grzeschik et al., 2010). Crumbs is a transmembrane protein with a large extracellular domain, and a short intracellular domain. Interestingly, the intracellular domain appears to mediate much of the functioning of Crumbs, as expression of only the intracellular domain coupled to a transmembrane domain is sufficient to rescue most of the phenotypes observed in *crumbs* mutant flies (Klebes and Knust, 2000; Wodarz et al., 1995). The intracellular domain contains a band 4.1 protein/Ezrin/Radixin/Moesin (FERM)-domain binding site and a C-terminal PSD-95/Discs large/ZO-1 (PDZ)-domain binding motif (Klebes and Knust, 2000). The PDZ-domain binding motif mediates binding to the Crumbs complex component Stardust/PALS1, as well as to Par6, and is essential for the establishment of cell polarity (Bulgakova and Knust, 2009; Klose et al., 2013; Morais-de-Sá et al., 2010; Walther and Pichaud, 2010). The FERM-domain binding motif mediates interactions with several FERM domain proteins, including Yurt, a negative regulator of Crumbs (Laprise et al., 2006), Expanded, an upstream component in the Hippo pathway (Ling et al., 2010; Robinson et al., 2010), the cytoskeletal protein βH-spectrin (Médina et al., 2002), and the cytoskeletal linker protein Moesin (Médina et al., 2002). The FERM-domain binding motif however is dispensable for polarity establishment in *Drosophila* embryonic epithelia (Klose et al., 2013).

Mammals have three Crumbs family members (CRB1-3) which all contain the conserved intracellular domain containing the FERM- and PDZ-domain binding motifs. However, CRB3 lacks the large extracellular domain present in the other family members and *Drosophila* Crumbs. Expression of *CRB1* in human and mice is limited to the retina and parts of the brain (den Hollander et al., 1999; den Hollander et al., 2002; van Rossum et al., 2006). Mutations in human *CRB1* cause retinitis pigmentosa (RP), while *Crb1* knockout mice show more limited retinal defects (den Hollander et al., 1999; van de Pavert et al., 2004). *CRB2* is expressed in the retina and kidney, while mouse *Crb2* is also broadly expressed during early embryonic development (van den Hurk et al., 2005; Xiao et al., 2011). Mice lacking *Crb2* die during gastrulation, likely due to disrupted polarity of epiblast cells, and conditional knockout of *Crb2* in the retina causes defects similar to RP (Alves et al., 2013; van de Pavert et al., 2004; Xiao et al., 2011). *CRB3* is broadly expressed in embryonic and adult epithelial tissues (Lemmers et al., 2004; Makarova et al., 2003; Yin et al., 2014). Knockdown and overexpression studies of CRB3 in MDCK cells, frog blastomeres, and human mammary cells indicate an important role for CRB3 in epithelial polarity establishment and junction formation (Chalmers et al., 2005; Lemmers et al., 2004; Roh et al., 2003; Schlüter et al., 2009; Whiteman et al., 2014). *Crb3* knockout mice die shortly after birth from epithelial defects, such as cystic kidneys and abnormal intestine with apical membrane blebs and disrupted microvilli (Whiteman et al., 2014).

In *C. elegans*, two Crumbs family members have been described: CRB-1 and EAT-20. CRB-1 localizes to the apical domain of intestinal and pharyngeal cells, starting in embryonic development (Bossinger et al., 2001; Segbert et al., 2004). In the embryonic intestine, CRB-1 localizes just apical of the junctional protein DLG-1 (Segbert et al., 2004). Loss of *crb-1* does not cause overt defects in polarity. However, an indication for a more subtle role in cell polarity for CRB-1 comes from studies examining the roles of the *C. elegans* Scribble homolog LET-413 and the *C. elegans* α-catenin homolog HMP-1 in positioning of DLG-1. Depletion of LET-413 results in disrupted positioning of DLG-1, while DLG-1 localization appears normal in *let-413 hmp-1* double knock down embryos and in *crb-1* knock down animals. Triple *let-413 hmp-1 crb-1* RNAi leads to a similar phenotype as *let-413* RNAi (Segbert et al., 2004). These results indicate a role for CRB-1 as a redundant mechanism for the correct positioning of DLG-1. EAT-20 is expressed in the pharynx, intestine, seam cells, a subset of neurons, and hypodermal cells (Achilleos et al., 2010; Shibata et al., 2000). A presumed null mutant of *eat-20* has a mild phenotype due to reduced pharyngeal pumping. The mutant worms have a starved appearance, a smaller brood size, and a prolonged egg-laying period (Shibata et al., 2000). *crb-1* and/or *eat-20* RNAi embryos develop normal epithelial identity (Bossinger et al., 2001; Segbert et al., 2004). Thus, although CRB-1 and EAT-20 localize apically, no essential role in polarity regulation has been uncovered for the Crumbs complex in *C. elegans*.

Here, we identify a third *C. elegans* Crumbs homolog, which is highly similar to mammalian CRB3 in size and domain structure. We show that this homolog of Crumbs is expressed in several polarized tissues in the embryo and larval stages and that the protein localizes apically in the intestine and pharynx. We used CRISPR/Cas9 technology to target all three Crumbs homologs for deletion, which did not result in apparent disruption of epithelial polarity. These results show that *C. elegans* contains an expanded Crumbs family consisting of three homologs, as is the case in mammals. However, the Crumbs complex does not appear to play an essential role in the establishment of epithelial polarity in *C. elegans*, and may instead contribute a more subtle or redundant function.

## MATERIALS AND METHODS

### Culture conditions and strains

*C. elegans* strains were maintained under standard culture conditions as previously described (Brenner, 1974). The wild-type strain used was Bristol N2. Unless otherwise indicated, strains were maintained at 15°C. The following strains were used: ST6: *eat-20(nc4)X*, BOX41: *mibIs23[lgl-1::GFP-Avi*, *Pmyo-3::mCherry]V*, BOX42: *mibIs24[crb-3::GFP-Avi*, *Pmyo-3::mCherry]IV*, BOX56: *mibIs31[dlg-1::GFP-Avi*, *Pmyo-3::mCherry]V*, BOX66: *mibIs41[crb-3::GFP-Avi*, *Pmyo-3::mCherry]III*, BOX51: *mibIs26[par-3::GFP-Avi*, *Pmyo-3::mCherry]V*, BOX142: *crb-1(mib3) eat-20(mib5) crb-3(mib4)X*, BOX143: *crb-3(tm6075)X*, BOX144: *mibIs31[dlg-1::GFP-Avi*, *Pmyo-3::mCherry]V; crb-1(mib3) eat-20(mib5) crb-3(mib4)X*, BOX145: *mibIs23[lgl-1::GFP-Avi*, *Pmyo-3::mCherry]V; crb-1(mib3) eat-20(mib5) crb-3(mib4)X*, BOX146: *mibIs26[par-3::GFP-Avi*, *Pmyo-3::mCherry]V; crb-1(mib3) eat-20(mib5) crb-3(mib4)X*

### Protein domain prediction and homology searches

To predict protein domains, we used the InterPro online prediction tool (http://www.ebi.ac.uk/interpro/) (Hunter et al., 2012). BLAST searches were performed through NCBI (http://www.ncbi.nlm.nih.gov/blast/Blast.cgi) and iterative HMMER searches were performed using the jackhammer online interface at Janelia Farms (http://hmmer.janelia.org/search/jackhmmer).

### Phylogenetic analysis

First, we identified candidate Crumbs proteins through an iterative HMMER search against the UniProtKB database. As the extracellular domain is highly variable in length and contains multiple EGF-like domains, which are present in a large number of proteins, we used the conserved transmembrane and intracellular domains for our search. The Human CRB1 C-terminal 73 amino acids were used as the starting sequence. From a list of homologous sequences obtained after 3 iterations of the search, we removed duplicate sequences. Next, the sequences were aligned using the online version of MAFFT with default settings (http://mafft.cbrc.jp/alignment/server/) (Katoh and Standley, 2013). From the aligned sequences, a phylogenetic tree was produced using the online version of PhyML with default settings (http://atgc.lirmm.fr/phyml/) (Guindon et al., 2010). Finally, the online Interactive Tree of Life tool was used to visualize the phylogenetic tree (http://itol.embl.de/) (Letunic and Bork, 2011). We rooted the resulting gene tree such that it minimizes the number of gene duplication events.

### Generation of GFP fusion constructs

To generate the GFP fusion constructs, we used the recombineering procedure previously described (Tursun et al., 2009). The sequences inserted by recombineering consist of *C. elegans* codon-optimized GFP containing FRT flanked GalK, derived from vector pBALU1 (Tursun et al., 2009), to which we added an Avi-tag (de Boer et al., 2003; Schatz, 1993) for future purification efforts. The GFP-Avi tag was inserted at the 3′ ends of the predicted genes. For *crb-3*, we amplified GFP-Avi using primers crb-3-F (5′-ACGCAAAAGACCTACCATATCTTCAACCTCCGAATGTAGAAGGACTTATCGGAGGGATCTGAGGAGGATCTGGAGGAGGA-3′) and crb-3-R (5′-CACATATAAAAGCGCCCAATTTGATTGAAATGAATAATAAAAATATTTTATTCATGCCATTCAATCTTCTGAGCTTCG-3′), for *dlg-1* we used primers dlg-1-F (5′-ACTCCATCATCAGCCGTGAATCGCAGACGCCAATTTGGGTGCCACGTCATGGAGGAGGATCTGGAGGAGGAGGATCTGGAGGAGGA-3′) and dlg-1-R (5′-ACATATTTCTTGAAGAAACGATTATTTGTCTAAAAAATATCCAATTTCATCTATTCATGCCATTCAATCTTCTGAGCTTCG-3′), for *lgl-1* we used primers lgl-1-F (5′- GAAGTACGGTGAATTTGAACTTTCGCGGTTGGAGCAGTACGCACAAGTCAGGAGGAGGATCTGGAGGAGGAGGATCTGGAGGAGGA-3′) and lgl-1-R (5′-AAAATTAATATATATCAACAGGAAAACGATTTTTAAAAAAAATGCATCTATTCATGCCATTCAATCTTCTGAGCTTCG-3′, and for *par-3* we used primers par-3-F (5′- GCCAATACCGTCGCAGAGATCAGGGACCGCCTCATCGTTTTCCCCAGTACGGAGGAGGATCTGGAGGAGGAGGATCTGGAGGAGGA-3′) and par-3-R (5′-GATTCCGTATTTTTCGCGGCTGCGTAATATAACTTTGAGAAAAAACTGACCTATTCATGCCATTCAATCTTCTGAGCTTCG-3′). Fosmids used were WRM0628dH07 carrying *crb-3*, WRM067dB05 carrying *dlg-1*, WRM065bB11 carrying *lgl-1*, and WRM064bG02 carrying *par-3*. All PCR amplifications were done with KOD hot start polymerase (Manufactured by Toyobo and distributed by Merck Millipore, Billerica, MA, USA) according to manufacturer's instructions, and an annealing temperature of 70°C. As an example of the final sequence, supplementary material Fig. S1 shows the final *crb-3::GFP-Avi* coding sequence.

### Generation of transgenic lines

Plasmid injections were performed using standard *C. elegans* injection procedures (Berkowitz et al., 2008). For γ–irradiation mediated integration, 100–150 late L4 stage animals carrying an extrachromosomal array transmitting at a rate of 20–60% were placed on a 6 cm NGM agar plate seeded with *E. coli* strain OP50. Next, a Cesium-137 source was used to deliver a dose of 4000 Gy of radiation. Following irradiation, animals were transferred to 9 cm NGM plates seeded with OP50, 10 animals per plate. Plates were allowed to starve for 7 days at 20°C. From each starved plate a large chunk (1/4 plate) was placed on a fresh seeded 9 cm NGM plate. After 1–3 days, 20 animals were transferred from each plate to individual 6 cm seeded NGM plates (200 animals total). After 4–5 days incubation at 20°C, plates were examined for 100% transmission rate. Integrated lines were backcrossed with N2 at least twice.

### CRISPR/Cas9

To generate deletion alleles of *crb-1*, *eat-20*, and *crb-3*, we simultaneously targeted a site near the start codon and a site near the stop codon of each gene with CRISPR/Cas9. To clone the sequences of the target sites into the sgRNA expression vector, we first annealed pairs of oligonucleotides crb-1_CRISPR_1_F (5′-AATTGACAATACACCTGGCTCTCT-3′) with crb-1_CRISPR_1_R (5′-AAACAGAGAGCCAGGTGTATTGTC-3′), crb-1_CRISPR_2_F (5′-AATTGAGAAAAGACACAGATGAAC-3′) with crb-1_CRISPR_2_R (5′-AAACGTTCATCTGTGTCTTTTCTC-3′), eat-20_CRISPR_1_F (5′-AATTGACAAAACTCCACTGAGAAA-3′) with eat-20_CRISPR_1_R (5′-AAACTTTCTCAGTGGAGTTTTGTC-3′), eat-20_CRISPR_2_F (5′-AATTGCTCGTGTACTCCCAAGTGA-3′) with eat-20_CRISPR_2_R (5′-AAACTCACTTGGGAGTACACGAGC-3′), crb-3_CRISPR_1_F (5′-AATTGAAAATGGCGTCAAACAGTA-3′) with crb-3_CRISPR_1_R (5′-AAACTACTGTTTGACGCCATTTTC-3′), and crb-3_CRISPR_2_F (5′-AATTGAATTAGTCTCGCTTTGCCT-3′) with crb-3_CRISPR_2_R (5′-AAACAGGCAAAGCGAGACTAATTC-3′). The resulting linkers were ligated into the BsaI digested U6::sgRNA expression vector pMB70 (Waaijers et al., 2013). For each deletion, we injected 30 animals with a mixture containing 5 ng/µl *Pmyo-3*::mCherry (pCFJ104, Addgene #19328), 50 ng/µl of each of the two sgRNAs and 50 ng/µl *Phsp-16.48*::Cas9 using standard *C. elegans* microinjection procedures. To induce expression from the *hsp-16.48* promoter, injected animals were heat shocked for 1 h at 34°C on agar plates floating in a water bath, 30 min after injection. From transgenic F1 animals expressing mCherry, we PCR amplified a region surrounding the target site using primers crb-1_CRISPR_check_F (5′-GTCGCTTGTTATGGGATAAAAC-3′) and crb-1_CRISPR_check_R (5′-GGTACCAGTGACAACATTTGCT-3′) for *crb-1*, eat-20_CRISPR_check_F (5′-GTGTGACCAAACTTATTGCTTTC-3′) and eat-20_CRISPR_check_R (5′-GCTCTCCAAGTCAAAAAGTTCTTA-3′) for *eat-20*, and crb-3_CRISPR_check_F (5′-GGAGACGGAGATGGTCAAGT-3′) and crb-3_CRISPR_check_R (5′-ACGTGTAGTACTCGGTGTTCAGG-3′) for *crb-3*. We established homozygous mutant lines by isolating single F2 animals and determining their genotype by PCR and sequence analysis. In addition to sequence analysis of each deletion, we verified that the deleted sequences had not inserted elsewhere in the genome using multiple sets of internal primers for each deletion (supplementary material Fig. S2). Internal primer sets used were crb-1_CF1 (5′-TTGCAGCCCATCTCTTCTTT-3′) and crb-1_CR1 (5′-CACTGAAACCCTTCGGACAT-3′), crb-1_CF2 (5′-GAGCGTCGAATGTTGTAGCA-3′) and crb-1_CR2 (5′-TTGCAGGTGCTAGAAGAGCA-3′), crb-1_CF3 (5′-GATTGAGAAAAACCGCGAAG-3′) and crb-1_CR3 (5′-ACACGATGACAACCGCAATA-3′), eat-20_CF1 (5′-GACCCCTCGGTTCTAGGAAG-3′) and eat-20_CR1 (5′-GGTGAAACCCTGACGACACT-3′), eat-20_CF2 (5′-GCCCAACACCAATGGTTATC-3′) and eat-20_CR2 (5′-CCACTCGAGGTGTGATGATG-3′), crb-3_CF1 (5′-GCATGGTTACTGAAGCGACA-3′) and crb-3_CR1 (5′-AACGTTTTCCCAGTTCCGTA-3′), crb-3_CF2 (5′-ATTACGGAACTGGGAAAACG-3′) and crb-3_CR2 (5′-GGTCTTTTGCGTGATGTTGTT-3′).

### Progeny counting and scoring of embryonic lethality

Starting at the L4 stage, individual animals were cultured at 20°C and transferred to a fresh plate every 24 h. Hatched and unhatched progeny were counted 24 h after removal of the P0.

### Embryo staining with MH27

For antibody staining, embryos were released from gravid adult animals by bleaching, and allowed to develop for 6 h at 20°C in M9 (0.22 M KH_2_PO_4_, 0.42 M Na_2_HPO_4_, 0.85 M NaCl, 0.001 M MgSO_4_). Embryos were then washed once in water, and a 10 µl drop of embryos was placed on a slide coated with poly-L-Lysine (Sigma-Aldrich Corp., St. Louis, MO, USA). Embryos were permeabilized by freeze-cracking, and fixed with methanol (5 minutes at −20°C) and acetone (20 minutes at −20°C). Embryos were stained on-slide as described (Duerr, 2006). Antibodies used were anti-AJM-1 MH27 mouse monoclonal supernatant (Developmental Studies Hybridoma Bank, Iowa City, IA, USA) diluted 1:20, and Alexa-Fluor 488 goat-anti-mouse (Life Technologies Europe, Bleiswijk, The Netherlands) diluted 1:500. Worms were mounted in Prolong Anti-Fade Gold (Life Technologies Europe, Bleiswijk, The Netherlands) supplemented with 2 µg/ml 4′,6-diamidino-2-phenylindole (DAPI; Sigma-Aldrich Corp., St. Louis, MO, USA).

### Microscopy and image processing

Microscopy of living animals was performed on a spinning disc platform consisting of a Nikon Ti-U inverted microscope with a motorized XY stage and a Piezo Z stage, 60× and 100× PLAN APO 1.4 NA oil objectives, a Yokogawa CSU-X1 spinning disk unit equipped with a dual dichroic mirror set for laser wavelengths 488 nm and 561 nm, 488 nm and 561 nm solid state 50 mW lasers controlled by an Andor revolution 500 series AOTF Laser modulator and combiner, Semrock 512/23 + 630/91 dual band pass emission filter, Semrock 525/30 single band pass emission filter, Semrock 617/73 single band pass filter, Semrock 4800 long pass filter (500–1200 pass), and an Andor iXON DU-885 monochrome EMCCD+ camera. All components are controlled by MetaMorph Microscopy Automation & Image Analysis Software. Microscopy of fixed samples was performed on a Zeiss LSM700 laser scanning confocal microscope equipped with a 63× Plan-Apochromat 1.4 NA objective, 405 nm, 488 nm, 555 nm, and 633 nm lasers, and the following emission filters: SP490 (400–490 nm), SP555 (400–555 nm), SP640 (400–640 nm), BP490-555 (490–555 nm), LP560 (560–750 nm), LP640 (640–750 nm) and BP592-662 (592–662 nm). The LSM700 is controlled by the Zen software package. All Z-stacks were taken with an 0.5 µm spacing, and maximum projections were generated with ImageJ. Final figures were produced using Adobe Photoshop CS6 and Adobe Illustrator CS6.

## RESULTS

### Identification of a candidate *C. elegans* CRB3 homolog

**Fig. 1.**
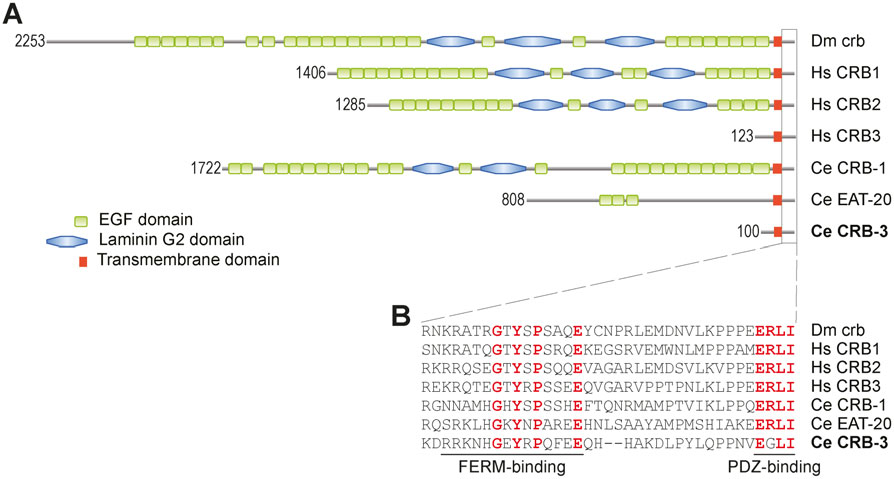
Homology between *Drosophila*, human, and *C. elegans* Crumbs proteins. (A) Protein domain structure of the Crumbs proteins. The number in front of the protein corresponds to the length of the protein in amino acids. (B) Intracellular part with conserved residues of the FERM-domain binding site and the PDZ-domain binding site depicted in red. The tyrosine and glutamic acid of the FERM-domain binding site were shown to be essential for *Drosophila* Crumbs (Klebes and Knust, 2000; Klose et al., 2013). Dm  =  *Drosophila melanogaster*, Hs  =  *Homo sapiens*, Ce  =  *Caenorhabditis elegans*.

Thus far two Crumbs homologs have been described in *C. elegans*: *crb-1* and *eat-20*. Both consist of a long extracellular region, a transmembrane (TM) domain, and a short intracellular region. CRB-1 is most similar in size and protein domain composition to *Drosophila* Crumbs (Fig. 1). CRB-1 consists of 1722 amino acids and contains 26 EGF repeats and two Laminin G-like domains in its extracellular region. The EAT-20 protein is 808 amino acids long and comprises three EGF repeats in its extracellular region. In both CRB-1 and EAT-20, the essential residues of the FERM-binding motif and the PDZ-domain binding motif are conserved in the intracellular region (Klebes and Knust, 2000; Klose et al., 2013). To identify potential additional Crumbs homologs, we searched the predicted *C. elegans* proteome for candidate homologs of Crumbs proteins by BLAST and HMMER. Searches with the human CRB3 sequence or intracellular domain of *Drosophila* Crumbs yielded a third significant hit, C35B8.4, in addition to CRB-1 and EAT-20. Large-scale expression profiling experiments indicate that the C35B8.4 gene is expressed (Levin et al., 2012; Spencer et al., 2011). The predicted protein encoded by C35B8.4 is 100 amino acids long, similar in length to mammalian CRB3, and consists of a short extracellular tail without recognizable domains, followed by a transmembrane domain and an intracellular part. The essential residues of the FERM-domain binding site and most residues of the PDZ-domain binding motif in the intracellular part are conserved (Fig. 1). A tyrosine at position 10 and a glutamic acid at position 16 of the intracellular part are part of the FERM-domain binding site and were shown to be essential for rescuing *crumbs* null phenotypes in *Drosophila* (Klebes and Knust, 2000; Klose et al., 2013). Both of these essential residues are conserved in *C. elegans* C35B8.4. The final four amino acids of CRB-3 are EGLI, and hence differ from the canonical ERLI PDZ-domain binding motif present in most Crumbs proteins. However, an alternative splice variant of human CRB3 also contains an alternative C-terminus (CLPI) and was shown to function in spindle assembly, cilia formation, and cell division. This alternative splice variant binds to importin β-1, unlike the ERLI isoform (Fan et al., 2007). Thus, the final four amino acids of C35B8.4 could potentially have a different binding specificity. In contrast to CRB-1, EAT-20, and the mammalian Crumbs proteins, C35B8.4 lacks a predicted N-terminal signal peptide. The transmembrane domain presumably acts as an internal ER signal sequence, and the positive charge of the residues following the transmembrane domain is consistent with a cytosolic C-terminus (Hartmann et al., 1989; Sipos and von Heijne, 1993). Phylogenetic analysis indicated that C35B8.4 is more similar to mammalian Crumbs3 proteins than to Crumbs1 or Crumbs2 proteins (supplementary material Fig. S3). Based on the similarity of C35B8.4 to human CRB3 and the apical localization of the protein described below, we assigned C35B8.4 the name *crb-3*.

### CRB-3 localizes apically in multiple polarized tissues

**Fig. 2.**
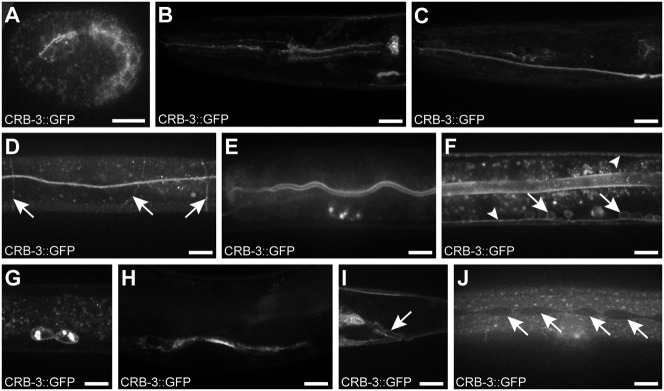
Expression and localization of CRB-3::GFP throughout development. (A) 1.5-fold embryo, (B–G,J) third larval stage, (H,I) fourth larval stage. (B) pharynx, (C) excretory canal, (D) circumferential pattern, indicated by arrows, resembling the pattern of commissural axons, (E) apical localization in the intestine (expression is also visible in a coelomocyte), (F) dorsal cord and ventral nerve cords indicated by arrowheads and cell bodies of the ventral nerve cord motor neurons indicated by arrows, (G) coelomocytes, (H) uterine epithelial cells, (I) rectal epithelium as indicated by the arrow, (J) seam cells indicated by arrows. Panels B and C show the head region of the same animal at different Z heights. Regions in panels D–F, H, and J are located along the middle of the body. Scale bars are 10 µm.

To determine a potential role for CRB-3 in establishing epithelial polarity, we first determined its expression pattern and subcellular localization. If CRB-3 acts as a regulator of epithelial polarity similar to Crumbs proteins in other organisms, we expect localization at the apical membrane domain of epithelial cells. To visualize the expression and localization pattern of CRB-3, we generated a translational CRB-3::GFP fusion. We made use of fosmid-based recombineering, to mimic the endogenous expression pattern as closely as possible (Tursun et al., 2009). We inserted the GFP-encoding sequence at the predicted 3′ end of the *crb-3* gene, and generated transgenic lines carrying an integrated copy of this construct by gamma-irradiation mediated integration of an extrachromosomal array. Two independently integrated strains showed the same expression pattern. CRB-3::GFP was first detected in embryonic pharyngeal and intestinal precursor cells (Fig. 2A). Throughout the larval stages the fusion protein localized to the apical membrane domain of pharyngeal cells, to the excretory canal, to the apical membrane domain of intestinal cells, to a circumferential pattern resembling the pattern of commissural axons, to neurons in the dorsal and ventral nerve cords, to the coelomocytes, and in a fraction of animals (n = 4/6) to the apical membrane domain of the rectal epithelium (Fig. 2). During the fourth larval stage, CRB-3::GFP became visible in the uterus (Fig. 2H). No fusion protein was detected in the seam cells or vulval epithelial cells, two tissues in which EAT-20 was shown to be expressed (Shibata et al., 2000) (Fig. 2H,J). The expression of CRB-3 in polarized tissues together with its apical localization in the pharynx and intestine strengthens our hypothesis that CRB-3 is a *bona fide* Crumbs homolog and third *C. elegans* Crumbs family member.

### The *C. elegans* Crumbs family is not essential for epithelial polarity

To investigate the role of *crb-3* in polarity establishment, we analyzed a deletion mutant (*tm6075*) obtained from the National Bioresource Project in Japan. This mutation is predicted to result in a frame shift and premature stop in *crb-3*. The corresponding protein lacks the transmembrane domain and intracellular region. Animals homozygous for the *tm6075* allele appeared healthy, and exhibited no embryonic or larval lethality. Examination of *crb-3(tm6075)* animals by Nomarski DIC microscopy also revealed no obvious developmental defects (not shown). These results indicate that *crb-3* is not essential for the establishment of epithelial polarity.

**Fig. 3.**
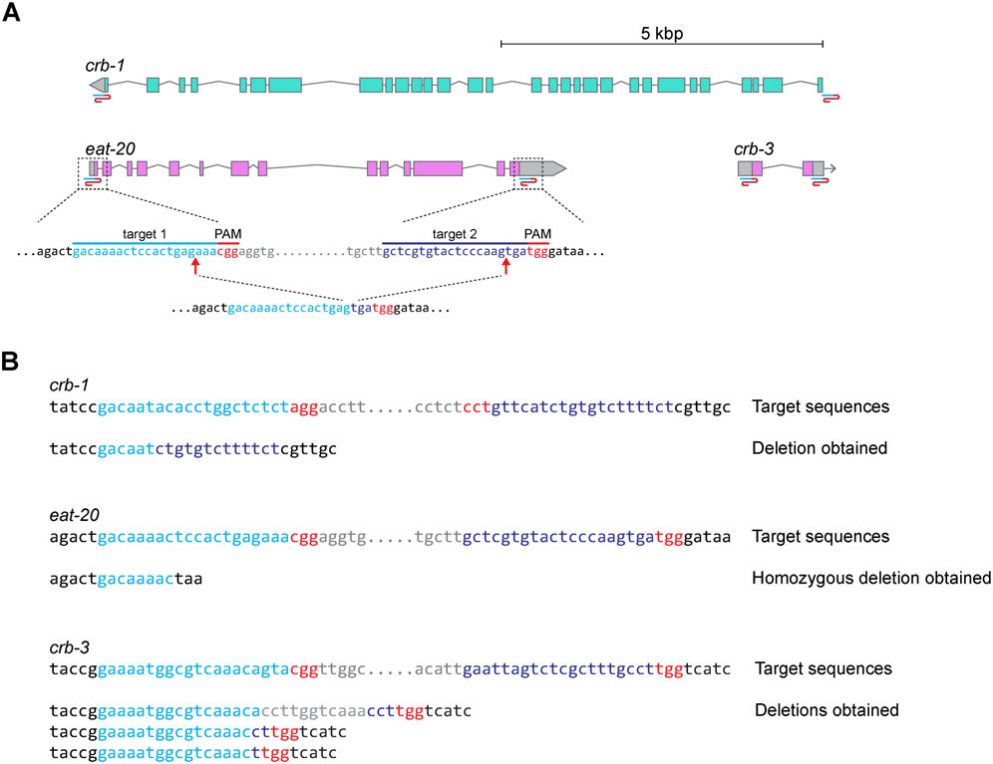
Generation of *crb-1*, *eat-20*, and *crb-3* deletions by CRISPR/Cas9. (A) Gene predictions with sgRNA target sites indicated by blue/red inverted S shape symbol. For *eat-20*, sequences of the target sites and expected deletion are shown. Genes on the forward strand are in pink, and on the reverse strand in blue. Gray boxes indicate untranslated regions. (B) Sequences of deletions obtained. Light blue  =  left Cas9 target site, dark blue  =  right Cas9 target site, red  =  PAM sequences, gray  =  sequence between targeted sites.

The expression pattern of *crb-3* shows extensive overlap with that of *crb-1* and *eat-20*, including expression of all three proteins in the intestine and pharynx of the developing embryo, and expression of at least *crb-3* and *eat-20* in larval tissues such as the pharynx, anal hypodermis, and coelomocytes (Bossinger et al., 2001; Shibata et al., 2000). One possible explanation for the limited defects we observed in *crb-3(tm6075)* animals and that were reported for *crb-1* and *eat-20* (Bossinger et al., 2001; Segbert et al., 2004; Shibata et al., 2000) is that functional redundancy exists between these genes. To investigate this possibility, we generated a strain lacking all three genes. For both *eat-20* and *crb-1*, deletion alleles exist as well. However, neither the *eat-20(nc4)* nor the *crb-1(ok931)* allele removes the entire gene coding sequences, and *crb-1(ok931)* is an in-frame deletion of part of the extracellular domain. Thus, it is possible that residual gene function remains due to *e.g.* alternative splicing, alternative start codons, or the remaining *crb-1* regions. To completely rule out the possibility of residual gene function, we decided to generate a triple knock-out strain in which we removed the entire predicted coding sequence of *crb-1*, *eat-20*, and *crb-3*. We used a CRISPR/Cas9-based approach to delete entire loci (Fig. 3). Previously, we used CRISPR/Cas9 to target a single DSB to specific loci in the genome, which results in the generation of small insertions or deletions due to errors during non-homologous end joining (Waaijers et al., 2013). By using two sgRNAs, one targeting a sequence before the start codon of the gene and the other targeting a sequence after the stop codon, the intervening sequence can be lost during DNA repair. Deletions of genes can easily be detected in the F1 generation by PCR with primers flanking the desired deletion. To generate a triple Crumbs knockout strain, we started from the *eat-20*(*nc4*) background, which has already lost part of *eat-20*. To delete the ∼11 kb *crb-1* coding sequence, we injected expression constructs for the two sgRNAs (*U6::sgRNA*), Cas9 controlled by the heat shock promoter (*Phsp-16.48*::Cas9) and a co-injection marker (*Pmyo-3::mCherry*) in the gonad of 30 P0 animals and exposed the injected animals to a 1 h heat shock at 34°C. We screened 89 transgenic F1 worms for deletion of the gene by PCR and obtained one deletion mutant. DNA sequence analysis confirmed the presence of a deletion with boundaries close to the predicted Cas9 cut sites, thus eliminating the entire *crb-1* coding region (Fig. 3B). The homozygous *eat-20 crb-1* double mutant did not show embryonic or larval lethality. Next, we used this double mutant as a background to delete the *crb-3* coding sequences using the same CRISPR/Cas9 approach. Out of 84 transgenic F1 worms, we obtained 3 *crb-3* deletion alleles that lacked the entire *crb-3* coding region. We sequenced the alleles and confirmed that each was a deletion with boundaries close to the predicted Cas9 cut sites (Fig. 3B). Finally, to ensure that no functional EAT-20 protein is produced, we deleted the remaining *eat-20* sequences, with a success rate of 13 deletion mutants out of 32 transgenic worms. We sequenced one allele that appeared to be homozygous in the F1 generation (Fig. 3B). We compared the brood size, egg laying period, and embryonic lethality of the homozygous triple *crumbs* deletion mutant with that of wild-type N2 animals and *eat-20(nc4)* animals. We observed no increase in embryonic lethality compared to N2 (supplementary material Fig. S4). As previously reported (Shibata et al., 2000), we observed a reduction in brood size and extension in egg laying period for *eat-20(nc4)* animals, which was not exacerbated in the *crb-1 eat-20 crb-3* triple deletion mutant (supplementary material Fig. S4).

**Fig. 4.**
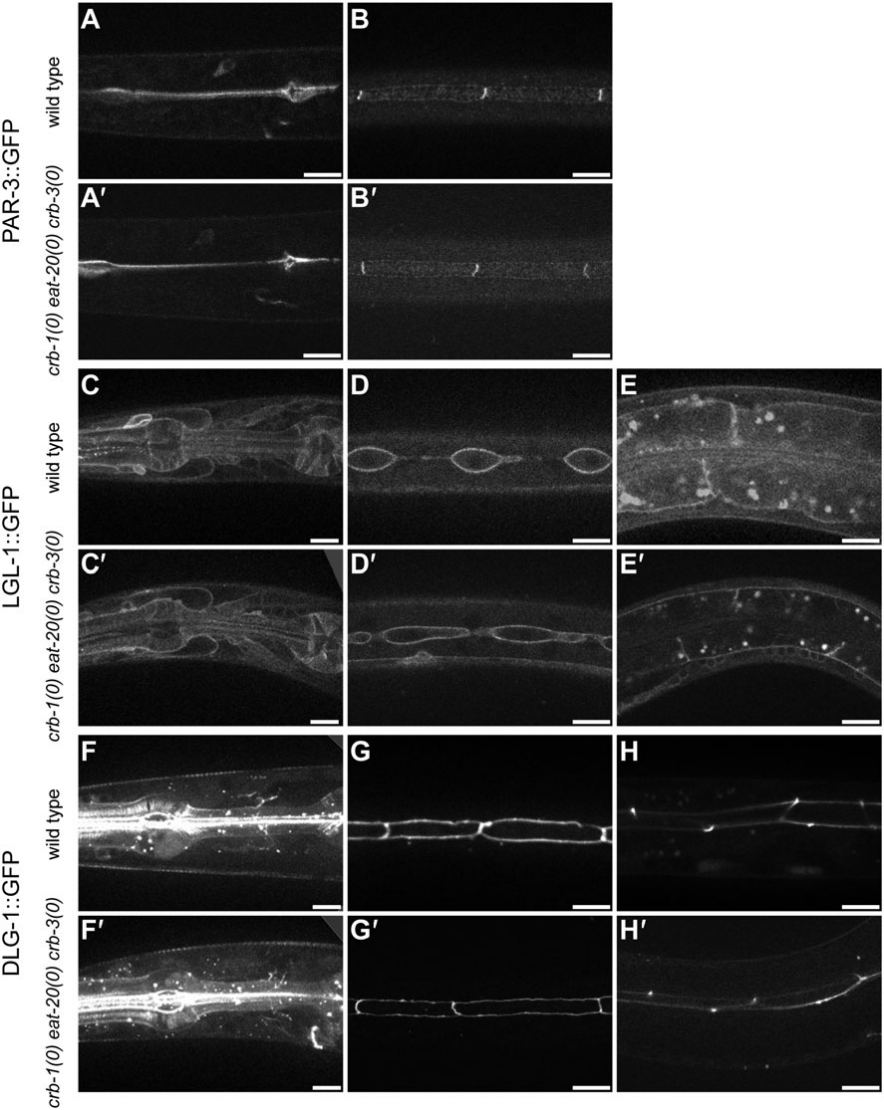
Expression of apical, basolateral, and junctional markers in wild type animals and the triple *crb-1 eat-20 crb-3* deletion strain. (A–H) wild type, (A′–H′) *crb-1(mib3) eat-20(mib5) crb-3(mib4)* triple deletion strain. (A,A′) pharyngeal expression of PAR-3::GFP, (B,B′) apical expression of PAR-3::GFP in the seam cells (confocal image taken at level of apical membrane), (C,C′) pharyngeal expression of LGL-1::GFP, (D,D′) basolateral expression of LGL-1::GFP in the seam cells (confocal image taken at level below the cell junctions), (E,E′) basolateral expression of LGL-1::GFP in the intestine, (F,F′) junctional localization of DLG-1::GFP in the pharynx, (G,G′) junctional localization of DLG-1::GFP in the seam cells, (H,H′) junctional localization of DLG-1::GFP in the intestine. Scale bars represent 10 µm.

**Fig. 5.**
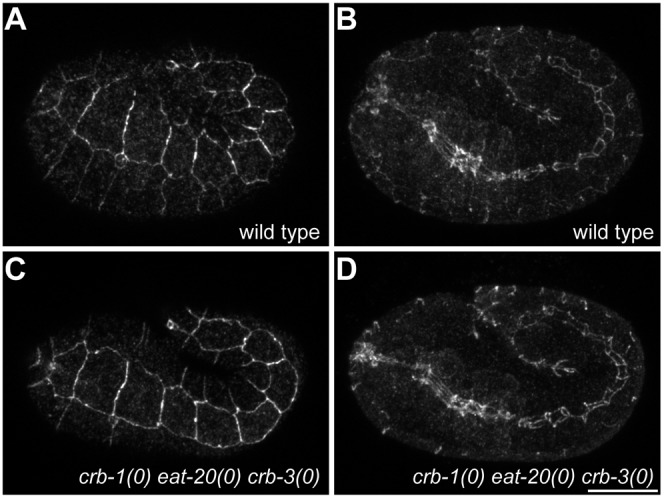
Localization of AJM-1 in wild-type and triple *crb-1 eat-20 crb-3* deletion embryos. Embryos were fixed and stained with the MH27 antibody, and imaged by confocal microscopy. All images are maximum intensity projections of slices taken 0.5 µm apart. (A,B) Wild-type 1.5-fold embryo. (C,D) *crb-1(mib3) eat-20(mib5) crb-3(mib4)* 1.5-fold embryo. (A,C) Projections of the outer 3 µm showing junctions between hypodermal cells. (B,D) Projections of the central 8 µm showing AJM-1 localization in the pharynx and intestine. Scale bars represent 10 µm.

To be able to analyze the effects of simultaneous loss of *crb-1*, *eat-20*, and *crb-3* on polarity in more detail, we created marker lines that express apically localized PAR-3::GFP, basolaterally localized LGL-1::GFP, or junctionally localized DLG-1::GFP. The expression constructs were generated by fosmid-based recombineering, and integrated into the genome by γ-irradiation. Each of the marker lines was crossed into the *crb-1(mib3) eat-20(mib5) crb-3(mib4)* triple deletion strain. The resulting strain was subsequently examined for effects of *crb*-family deletion on localization of polarity proteins in larval epithelia. For LGL-1 and DLG-1, we examined the pharyngeal epithelium, seam cells, and intestine. For PAR-3, which was not expressed in the intestine, we examined the pharyngeal epithelium and seam cells. In all cases, the localization of the GFP-tagged polarity proteins was similar to the wild type localization pattern (Fig. 4). Because of the previously described possible contribution of *crb-1* to junction assembly (Segbert et al., 2004), we also examined the formation of apical junctions in the *crb-1(mib3) eat-20(mib5) crb-3(mib4)* triple deletion mutant strain. To visualize apical junctions, we stained 1.5-fold embryos with the MH27 antibody, which recognizes the junctional component AJM-1. We compared the MH27 staining pattern in the triple mutants to the characteristic pattern of cell junctions in wild-type embryos and, again, observed no abnormalities (Fig. 5, compare A to C and B to D). Taken together, our analysis of a triple *crumbs* deletion mutant indicates that the function of the three Crumbs family members is not critical for establishment of apical-basal polarity in *C. elegans* epithelia.

One possibility for the observed lack of phenotype in the triple *crumbs* deletion strain is that *C. elegans* Crumbs proteins function redundantly with other polarizing mechanisms. To investigate this possibility, we inactivated *cdc-42*, *par-3*, *par-6*, *pkc-3*, *hmr-1*, *hmp-2*, *let-413* or *lgl-1* by RNAi in the triple *crumbs* deletion strain. Feeding RNAi was started at the L4 stage, and we counted the number of hatched and unhatched embryos produced 0–8 h, 8–32 h, and 32–56 h after the start of RNAi. Inactivation of *pkc-3* and *let-413* resulted in 100% embryonic lethality, precluding observation of any synergistic effect of the triple *crumbs* deletion (Table 1). RNAi for *cdc-42*, *par-6*, *hmr-1* resulted in partial lethality, while *lgl-1* RNAi did not induce embryonic lethality. Additional inactivation of the *C. elegans crumbs* family did not result in increased embryonic lethality for any of these genes. Finally, RNAi for *par-3* and *hmp-2* resulted in a very limited number of hatched progeny produced in the first 32 h after the start of RNAi feeding. For both genes, additional inactivation of the *crumbs* genes reduced the fraction of hatched progeny produced in this time period. However, the already small number of hatching progeny in the wild-type background makes it difficult to draw a firm conclusion regarding a redundant function of *par-3* or *hmp-2* with the *crumbs* genes.

**Table 1.**
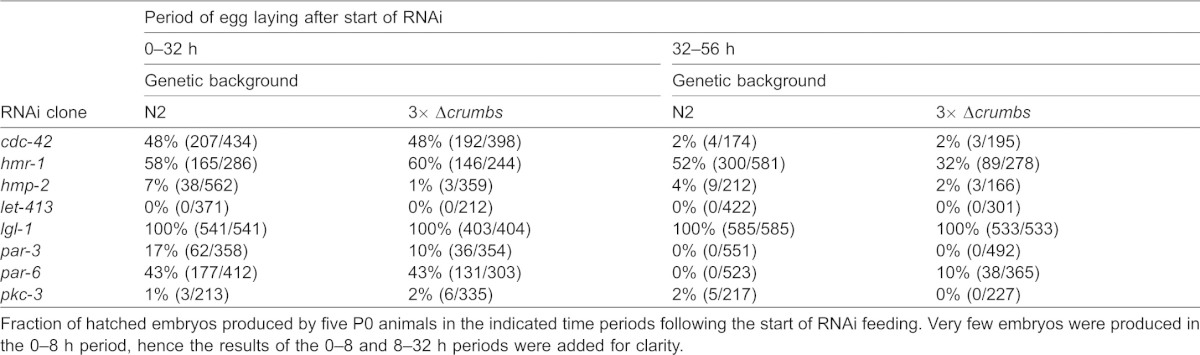
Embryonic survival after RNAi in wild type or 3× Δ*crumbs* background

## DISCUSSION

Here, we identified a third *C. elegans* Crumbs family member, which we termed CRB-3, based on similarity to mammalian CRB3. Using a translational CRB-3::GFP fusion, we observed expression of *C. elegans crb-3* in several polarized tissues in the embryo and in larval stages, with clear apical localization of CRB-3::GFP in the intestine and pharynx. Our results show that the Crumbs family in *C. elegans* consists of at least three members, similar as in mammals and in contrast to the single *Drosophila crumbs* gene. We deleted all three *C. elegans* Crumbs homologs using a CRISPR/Cas9-based approach. Surprisingly, given the importance of the Crumbs family in other organisms, animals that lack all three *crumbs* homologs were viable and showed no more severe defects than the starved appearance, reduced brood size, and extended egg laying period previously described for *eat-20(nc4)* (Shibata et al., 2000). Moreover, localization of PAR-3::GFP, LGL-1::GFP, or DLG-1::GFP was unaffected in the triple *crumbs* homolog deletion strain. These results show that, despite evolutionary conservation, the Crumbs family has no essential role in *C. elegans*.

One possible explanation for the observed lack of a phenotype is that the *C. elegans* Crumbs proteins control specific aspects of epithelial cells, rather than critically regulating apical polarity. For example, CRB-1, EAT-20, or CRB-3 may act through FERM-domain containing proteins like ERM-1 or SMA-1, the *C. elegans* homologs of Moesin and βH-Spectrin respectively, to contribute to the regulation of the actin cytoskeleton in tissues like the intestine or excretory canal. Subtle defects in these tissues would not have been uncovered using the approaches employed here. Alternatively, the *C. elegans* Crumbs proteins may function redundantly with other polarizing mechanisms. In support of this hypothesis, a previous study observed that CRB-1 can provide a positional cue for the localization of DLG-1 after inactivation of the basolateral regulator *let-413* Scribble and the junctional component *hmp-1* α-Catenin (Segbert et al., 2004). Thus, LET-413 and the Cadherin/Catenin complex (CCC) are likely candidates for a redundant mechanism. Another possible candidate for acting redundantly with the Crumbs proteins is the apical PAR complex, which is known to act together with Crumbs to establish apical identity in *Drosophila* (Krahn et al., 2010; Walther and Pichaud, 2010). We investigated potential redundancy by examining if inactivation of PAR or CCC components by RNAi caused enhanced embryonic lethality in the triple *crumbs* deletion strain. We did not observe a synergistic effect between deletion of the Crumbs family and inactivation of the PAR complex component PAR-6, or the CCC component HMR-1. Similarly, we observed no redundancy between the Crumbs family and CDC-42 or LGL-1. We were not able to extend this analysis to PAR-3, PKC-3, LET-413, or HMR-1, as RNAi for the corresponding genes already causes extensive embryonic lethality in the wild-type background. Thus, it remains possible that the Crumbs family acts redundantly with these, or other polarity regulators.

The composition of the *C. elegans* Crumbs complex has not been further investigated to date. The core Crumbs complex in *Drosophila* consist of Crumbs, Stardust (PALS1 in mammals), PATJ, and Lin-7 (Bulgakova and Knust, 2009). Of these, only Crumbs and Stardust are broadly required for epithelial polarity, while PATJ and Lin-7 have more specific functions (Bachmann et al., 2008; Nam and Choi, 2006). The *C. elegans* genome encodes three candidate Stardust homologs: MAGU-1, MAGU-2, and MAGU-3 (Assémat et al., 2008; Knust and Bossinger, 2002), of which MAGU-2 is most similar to PALS1 and Stardust. The subcellular localization pattern of these proteins has not been determined. The likely null allele *magu-2(gk218)* is reported to be homozygous viable. No good candidate homolog of PATJ exists in *C. elegans*. The closest homolog, MPZ-1, resembles both PATJ and MPDZ/MUPP1 in that it contains a high number of PDZ domains. However, MPZ-1 lacks the characteristic L27 domain present in PATJ and Stardust, and functional analysis of MPZ-1 suggests that it is more likely to represent a homolog of MPDZ (Xiao et al., 2006). Finally, LIN-7 was originally identified in *C. elegans*, where it acts in a complex with LIN-2 and LIN-10 to control the basolateral localization of the EGF receptor LET-23 in vulval epithelial cells (Kaech et al., 1998; Simske et al., 1996). A potential role for LIN-7 as a component of a Crumbs complex has not been investigated.

Together with the PAR and Scribble groups, the Crumbs complex regulates cell polarity in a variety of different epithelial cell types. However, it is clear that the mechanisms through which these evolutionarily conserved proteins establish polarity vary markedly in different cell types or conditions. In *Drosophila*, not all epithelia in which Crb is expressed require Crb to maintain epithelial polarity (Tepass, 2012). Similarly, even though mouse *Crb3* is widely expressed in embryonic tissues, *Crb3* knockout mice complete embryogenesis and die shortly after birth (Whiteman et al., 2014). During development of the *Drosophila* embryo, at least three groups of basolateral regulators function at different times (Tepass, 2012). In the *C. elegans* embryo, PAR-3 is required for the assembly of cell junctions in intestinal cells, but apical junctions still form in the absence of PAR-3 in epidermal epithelia (Achilleos et al., 2010). It is important therefore to study the functioning of polarity regulators in a range of different systems and organisms. Though it remains unclear what the exact role of the Crumbs proteins is in *C. elegans*, our identification of a Crumbs3 homolog provides further insight into the *C. elegans* Crumbs family.

## Supplementary Material

Supplementary Material
