# Supplementary material for: The C. elegans Crumbs family contains a CRB3 homolog and is not essential for viability
Source: Biol Open. 2015 Feb 6;4(3):276–84. doi: 10.1242/bio.201410744 (PMC4359734; doi:10.1242/bio.201410744)
Supplement: Supplementary Material [file supp_4_3_276__index.html]

The C. elegans Crumbs family contains a CRB3 homolog and is not essential for viability — The C. elegans Crumbs family contains a CRB3 homolog and is not essential for viability — Supplementary Material 

# The *C. elegans* Crumbs family contains a CRB3 homolog and is not essential for viability

## bio.201410744 Supplementary Material

**Files in this Data Supplement:**

- Supplementary Material - Selma Waaijers et al. doi: 10.1242/bio.201410744
