# Supplementary material for: The C. elegans Crumbs family contains a CRB3 homolog and is not essential for viability
Source: Biol Open. 2015 Feb 6;4(3):276–84. doi: 10.1242/bio.201410744 (PMC4359734; doi:10.1242/bio.201410744)
Supplement: Supplementary Material [file supp_bio.201410744_bio.201410744-s1.pdf]

Biology Open (2015) 000, 1–9 doi:10.1242/bio.201410744

Supplementary Material

Selma Waaijers et al. doi: 10.1242/bio.201410744

```
ATGGCGTCAAACAGTACGGTTGGCATGGTTACTGAAGCGACAGTTTCTTTGATAGAAGCTGTCAGTCAGGAAGCCGTG
GAAGCATCAAACAAAGGTGGTGGATTATCTGGTGGAGCAATTGCGGGGATTGTCATTGCAGTAGTTgtgagtcattta
aaaacatgggttgaactctgaatgagttctgaaaatgtgtgttaaaccaattatttaatgttgactcacaagttaaat
atcatatatatcttgcgttgaaagataggtgaagtaaatttgaaaatatttttttgaaccttcaaaactaaacagtctc
aaaaaatgtaatacagaaataggtttaagacactgataaaaattatggacaacatatattttaacaaacctctcgagc
ttaatgtttattatgttcagtaaattacggaactgggaaaacgttttcttaaaaaatattagctttaaaatttgttcg
tttttcgaacaagtttgaactaatttcccaacttgtatattataaattgatttaattatttctttatgagtaaattat
ttaacggttactataaaatatattaatgtttcgttttgtagttattttgggtagatgtatgagcgttatacatacata
aacgtttgtcgccaaattcaattgccaattgatttttgaattcctgataatataatttttagaatcttaataacacgc
acggaatacctaaagaatacagttgaataaaatttgcatattactacagATTGGAGTGCTCCTGATTGGAGTCGTCGG
CTTCTTTGCATTCAAATATGTGAAAGATAGACGAAAAAACCATGGTGAATACCGTCCACAATTTGAAGAACAACATCA
CGCAAAAGACCTACCATATCTTCAACCTCCGAATGTAGAAGGACTTATCGGAGGAGGATCTGGAGGAGGAGGATCTGG
AGGAGGAGGAATGAGTAAAGGAGAAGAACTTTTCACTGGAGTTGTCCCAATTCTTGTTGAATTAGATGGTGATGTTAA
TGGGCACAAATTTTCTGTCAGTGGAGAGGGTGAAGGTGATGCAACATACGGAAAACTTACCCTTAAATTTATTTGCAC
TACTGGAAAACTACCTGTTCCATGGgtaagtttaaacatatatatactaactaaccctgattatttaaattttcagCC
AACACTTGTCACTACTTTCTGTTATGGTGTTCAATGCTTCTCGAGATACCCAGATCATATGAAACGGCATGACTTTTT
CAAGAGTGCCATGCCCGAAGGTTATGTACAGGAAAGAACTATATTTTTCAAAGATGACGGGAACTACAAGACACgtaa
gtttaaacagttcggtacgaagttcctatactttctagagaataggaacttctaactaaccatacatatttaaatttt
cagGTGCTGAAGTCAAGTTTGAAGGTGATACCCTTGTTAATAGAATCGAGTTAAAAGGTATTGATTTTAAAGAAGATG
GAAACATTCTTGGACACAAATTGGAATACAACTATAACTCACACAATGTATACATCATGGCAGACAAACAAAAGAATG
GAATCAAAGTTgtaagtttaaacatgattttactaactaactaatctgatttaaattttcagAACTTCAAAATTAGAC
ACAACATTGAAGATGGAAGCGTTCAACTAGCAGACCATTATCAACAAAATACTCCAATTGGCGATGGCCCTGTCCTTT
TACCAGACAACCATTACCTGTCCACACAATCTGCCCTTTCGAAAGATCCCAACGAAAAGAGAGACCACATGGTCCTTC
TTGAGTTTGTAACAGCTGCTGGGATTACACATGGCATGGATGAACTATACAAATCTGGAGGAGGATCTGAAAATCTTT
ATTTCCAAGGATCCGAGAACCTTTACTTCCAAGGATCTGGAGGAGGATCTGGACTTAATGATATTTTCGAAGCTCAGA
AGATTGAATGGCATGAATAA
```

crb-3

flexible linker

GFP

FRT site

2 x TEV site

Avi tag

Fig. S1. Sequence of CRB-3 tagged with the GFP-Avi cassette.

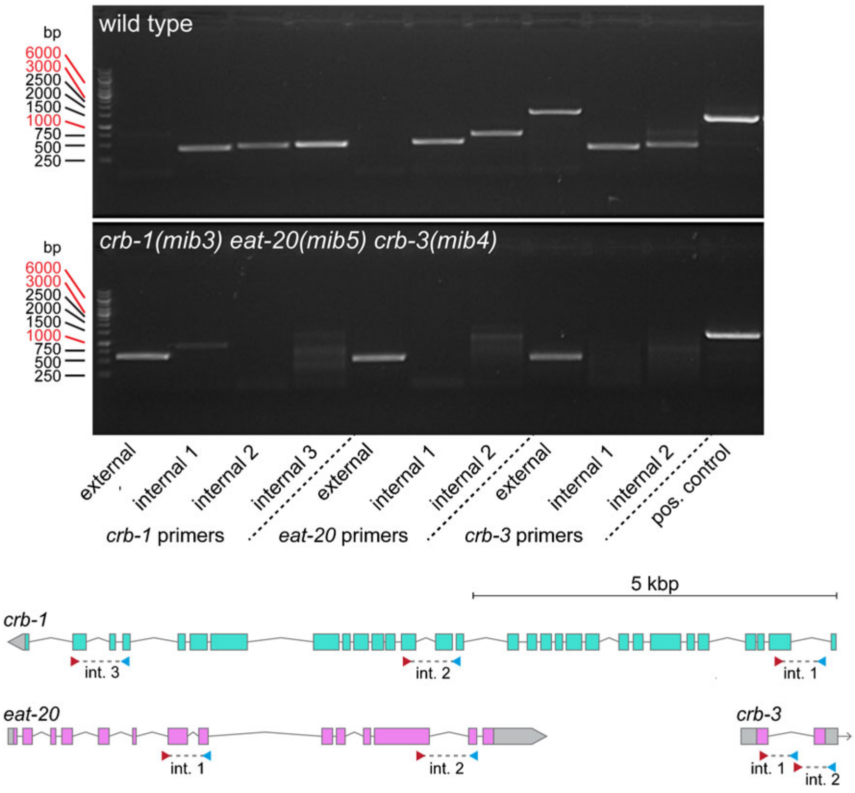

Fig. S2. PCR analysis of the triple crumbs family deletion strain. Wild-type (N2) and *crb-1(mib3) eat-20(mib5) crb-3(mib4)* animals were analyzed by PCR amplification using primers external to the coding sequence of each gene, as well as several internal primer sets. Due to the large gene sizes, with the PCR conditions used the external primer sets for *crb-1* and *eat-20* are expected to yield an amplicon only when the gene is deleted. Internal primer sets should result in an amplicon in the N2 strain, but not in the triple deletion strain. The locations of the internal primer sets are indicated in the graphical representation of the genes (forward primers in red, reverse primers in blue). Genes on the forward strand are in pink, and on the reverse strand in blue. Grey boxes indicate untranslated regions. A single lysate was used for all PCR reactions, and a positive control was included to show the suitability of the lysate for PCR amplification.

Biology Open (2015) 000, 1–9 doi:10.1242/bio.201410744

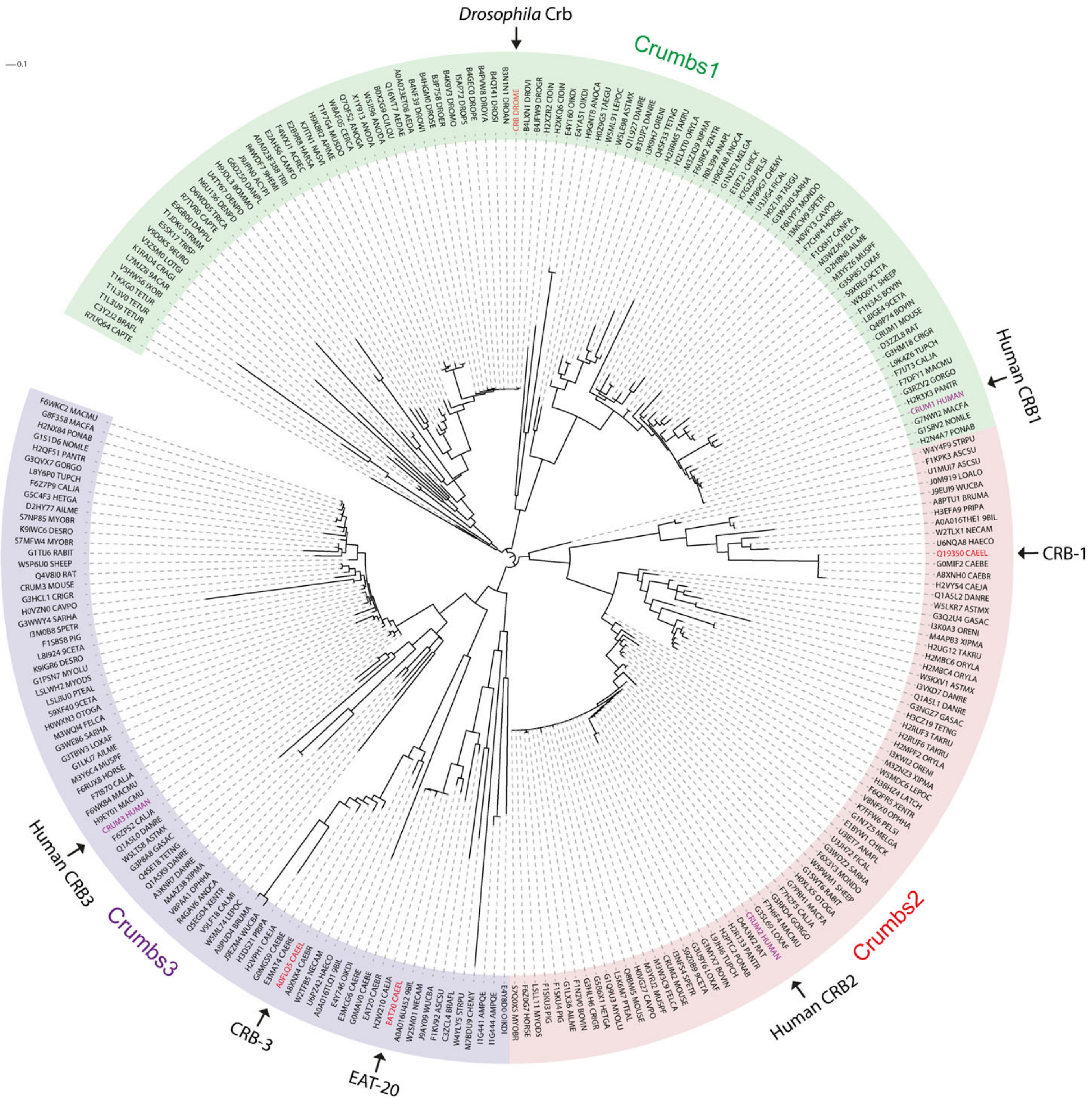

Fig. S3. Phylogenetic tree of Crumbs homologs. Proteins were identified through iterative HMMER searches as implemented in JACKHMMER with the transmembrane and intracellular domains of human CRB1. Three major groups containing the human CRB1, CRB2, and CRB3 proteins are color coded. Arrows indicate Crumbs proteins in Human, *C. elegans*, and *Drosophila*. All names are UniProt IDs. Scale bar represents 0.1 substitutions per residue.

Biology Open

Biology Open (2015) 000, 1–9 doi:10.1242/bio.201410744

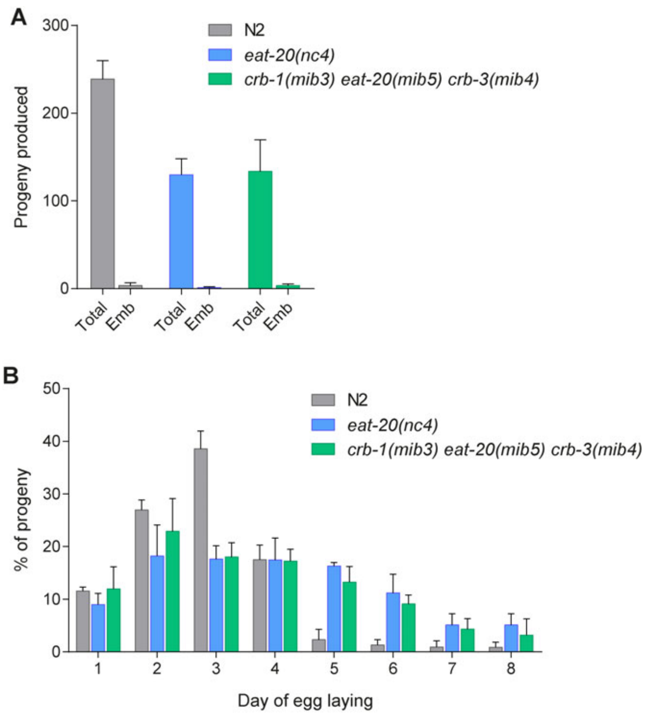

Fig. S4. *eat-20(nc4)* and *crb-1(mib3) eat-20(mib5) crb-3(mib4)* cause a similar reduction in brood size and extension of egg laying period.
(A) Progeny produced by N2, *eat-20(nc4)*, and *crb-1(mib3) eat-20(mib5) crb-3(mib4)* animals. Total: average total progeny produced. Emb: average number of embryonic lethal progeny produced. (B) Fraction of progeny produced on 8 consecutive 24 h periods. Bars represent average values, and error bars the standard deviation. For N2, n=5; for *eat-20* and *crb-1 eat-20 crb-3*, n=4.

Biology Open
